# Neurocognitive variability in schizophrenia spectrum disorders: relationship to real-world functioning

**DOI:** 10.1016/j.scog.2020.100172

**Published:** 2020-02-14

**Authors:** Lars Helldin, Christine Mohn, Anna-Karin Olsson, Fredrik Hjärthag

**Affiliations:** a Department of Psychiatry, NU Health-Care Hospital, Region Västra Götaland, Sweden; b Department of Psychology, Karlstad University, Sweden; c NORMENT, Institute of Clinical Medicine, University of Oslo, Norway

**Keywords:** Functional outcome, Neurocognition, Real-world functioning, Psychosis, Schizophrenia

## Abstract

Neurocognitive variability exists within the schizophrenia spectrum disorder (SSD) population, with subgroups performing at the same level as healthy samples Here we study the relationship between different levels of neurocognitive responding and real-world functioning. The participants were 291 SSD patients and 302 healthy controls that were assessed with a comprehensive neurocognitive battery. In addition, the patients were assessed with the Specific Level of Functioning Scale (SLOF). The results showed that the mean neurocognitive test responses of the SSD group were significantly below that of the control group. However, there was considerable overlap between the cognitive scores of the two groups, with as many as 24% of the patients performing above the mean healthy score for some domains. Moreover, the patients with the highest level of neurocognitive functioning reached the highest levels of practical and work-related functioning outcome skills. There was no significant relationship between neurocognitive and social function skills. The large differences in cognitive performance and their associations with functional outcome within the patient group are rarely addressed in clinical practice, but indicate a clear need for individualized treatment of SSD. Early identification of cognitive risk factors for poor real-life functional outcome is necessary in order to alert the clinical and rehabilitation services about patients in need of extra care.

## 1 Introduction

Neurocognitive dysfunction is a hallmark of schizophrenia spectrum disorders (SSD) (Kahn and Keefe, 2013), is relatively stable across time (Rund et al., 2016), and is related to poor real-world functional performance (Bowie and Harvey, 2006). However, within the SSD population, there is considerable neurocognitive diversity, with subgroups performing at the same level as healthy samples (Fioravanti et al., 2005; Wilk et al., 2004). Differences in cognitive profile are relevant for clinical and functional outcomes. Poor cognitive functioning is related to worsened treatment response (Kennedy et al., 2015) and vocational ability (Lystad et al., 2016). The clinical community has generally failed to address the individual neurocognitive differences in the SSD group. This is unfortunate, as the functional outcome differences in SSD persist even though psychosis symptom remission is achieved, as evidenced by the low rates of completely independent living and work force participation among the SSD population. Tailoring psychosis treatment to in-group neurocognitive variability could reduce the differences in real-life outcome.

One aim of the Clinical Longterm Investigation of Psychosis in Sweden (CLIPS) project is the identification of individual differences predicting outcome differences. We have previously demonstrated that SSD patients with poor neurocognitive function are less likely to obtain symptom remission (Helldin et al., 2006), more likely to develop poor physical functioning and somatic ill health (Moradi et al., 2018), and to die prematurely (Helldin et al., 2015) compared to cognitively intact peers.

Here, we extend this research into the functional outcome field. This topic is not new, and others have reported associations between poor neurogocnitive function and real-life skills in SSD patients. Kurtz and Wexler (2006) demonstrated significant differences in functional capacity based on differences in executive function. Differences in verbal learning predicted the results of everyday life skills rehabilitation (Kurtz et al., 2008). Fu et al. (2017) found that low attention, verbal learning, and working memory scores at baseline predicted low levels of social function 4 years later. Straussnig et al. (2015) reported significant associations between general neurocognitive function (i.e., a composite score) and several types of practical skills, but not social function.

A common limitation of most of these studies is a relatively low sample size. Moreover, these projects have employed samples that are predominately male (Kurtz and Wexler, 2006; Kurtz et al., 2008; Harvey et al., 2011, Harvey et al., 2013; Heinrichs et al., 2010; Reichenberg et al., 2014; Strassnig et al., 2015). In addition, most studies employ mostly in-patients or a combination of inpatients and outpatients. Outpatients should be the most relevant group for real-world function studies, as they are expected to manage mostly on their own.

Here we analyze data from a large, gender mixed, and population based sample of outpatients who live independently or semi-independently in the general community. Moreover, we employ the Specific Level of Functioning Scale (SLOF) (Schneider and Struening, 1983) for assessments of real-world functioning. This scale has two major advantages compared to others. First, it is free of cognitive items that may confound the neurocognitive-functional outcome relationship. Second, it has been identified as the self-rating scale with the closest relationship to everyday functioning (Harvey et al., 2011), and has been used in a previous relevant study with less strict inclusion criteria (Strassnig et al., 2015).

In summary, we have the following aims of the present study: (1) To describe the overlap of neurocognitive scores of SSD patients and healthy controls, and (2) to describe the relationship between neurocognitive diversity and real-world functioning in the patient group. To the best of our knowledge, this is the first study to do so in a gender-balanced sample of outpatients. This paper will lay the groundwork for future studies of individualized treatment and rehabilitation strategies based on personal differences in core clinical variables in our patient group.

## 2 Materials and methods

This study is part of the CLIPS project, which has been described in detail elsewhere (Helldin et al., 2015). Briefly, men and women suffering from SSD were recruited from the outpatient clinics in the NU Health Hospital region in South-Western Sweden from November 2000. Two thirds of all eligible patients on this geographic catchment area were included at baseline. The participants were required to be in a stable clinical condition and to be suffering from no other illness than psychosis upon inclusion. Patients who reported significant past or present use of alcohol or illegal substances were excluded. Each participant gave their written informed consent to participation. The project was approved by the Research Ethics Committee in Gothenburg, Sweden, (approval number: Ö537–99, 507–04, 438–10, 423–14) and carried out according to the Helsinki Declaration.

### 2.1 Participants

The patient group consisted of 291 individuals (Table 1). Most of them suffered from schizophrenia (*n* = 188, 64.6%) or schizoaffective disorder (*n* = 69, 23.7%), while 34 (11.7%) was diagnosed with delusional disorder. The vast majority were native Swedish speakers, and 5 required the assistance of an interpreter during the assessments.

**Table 1:** Demographic and clinical characteristics of the participants.

	SSD patients (*n* = 291)	Healthy controls (*n* = 302)
Gender	164 (56.4%) men	123 (40.7%) men
	127 (43.6%) women	179 (58.9%) women
Age	46.7 (12.3), range 19–83	47.8 (17.7), range 19–80
Highest level of education		
Elementary school	126 (44.1%)	60 (19.9%)
Senior high school	114 (39.9%)	72 (24.5%)
College level	45 (15.7%)	168 (55.6%)
Marital status		
Single (never married)	163 (56.2%)	62 (20.5%)
Married/partnered	68 (23. %)	179 (59.3%)
Divorced/widowed	59 (20.3%)	60 (19.9%)
Duration of illness	20.4 (11.6)	–
Hospitalizations	7.2 (7.9)	–
In remission	114 (39.2%)	–
PANSS		
Positive	12.0 (4.8)	–
Negative	16.1 (5.2)	–
General	30.8 (7.1)	–
Total	58.8 (13.8)	–

Age, GAF, duration of illness, number of hospitalizations, and PANSS in mean (SD). Some of the percentages do not add up to 100, as there are missing data for a small number of participants.

The healthy controls were 302 individuals recruited as part of a psychometric standardization study at Karlstad University. Exclusion criteria were ongoing mental or severe somatic illness, substance abuse, mental retardation, a history of neurological insults or injury, and lack of fluency in Swedish.

### 2.2 Assessments

Diagnosis was determined according to the DSM-IV (APA, 1994) and ISD-10 (WHO, 1993) criteria. Functional level was determined according to the Global Assessment of Function (GAF), and the total score was split into a symptom and function sub score (Pedersen et al., 2007). Positive, negative, and general symptoms of schizophrenia were assessed with the Positive and Negative Syndrome Scale (PANSS) (Kay et al., 1987). Remission status was established according to the guidelines of Andreasen et al. (2005). Real-life functioning was assessed with the SLOF (Schneider and Struening, 1983), consisting of 43 items that tap 6 subscales. The items are scored on a Likert scale anchored at 1 (poorest function) and 5 (best function), depending on the frequency during the last week. The SLOF was rated by each individual patients' case worker.

After the clinical assessment, the participants performed the following neurocognitive tests in their Swedish versions:

The *Trail Making Test A* ( *TMT-A*) *and B* ( *TMT-B*) (Reitan, 1958) were used as measures of visuomotor speed (part A) and cognitive flexibility (part B). The participants are asked to join consecutive numbers (part A) or alternate between two sets by connecting letters and numbers (part B) as rapidly as possible.

The *Letter Number Span test* ( *LNS*) (Gold et al., 1997) was used as a measure of auditory working memory and requires the participant to order and repeat random combinations of letters and numbers read aloud by the administrator.

The *Rey Auditory Verbal Learning Test* ( *RAVLT*) (Schmidt, 1996) consists of a 15-item word list that is read aloud to the participant five times, with an assessment of recall immediately after each presentation. Then, an interference list is presented, followed by the request to recall the original list of words. Finally, a delayed recall test is presented after 20 min. In this study, we used the number of words correctly recalled in trial 1 to trial 5 (RAVLT 1–5 sum) as an indication of learning, and the number of words correctly recalled after 20 min (RAVLT 7) as an indication of retention memory.

The *Continuous Performance Test – Identical Pairs* ( *CPT-IP*) (Cornblatt et al., 1988) is a computerized test assessing attention and vigilance. In this study, only the numbers part was used. The participants monitor 4-digit numbers on a computer screen and press a button when two identical combinations of digits are presented in a row.

The *Wisconsin Card Sorting Test* ( *WCST*) (Heaton et al., 1993) is a computerized test of executive function. It consists of four stimulus cards and 128 response cards that depict figures, colors, and numbers. The participants match each consecutive card from the deck with one of the stimulus cards by pressing a computer key. Once the participants have made a specified number of consecutive correct matches to the initial sorting principle, the sorting principle changes without warning. The test proceeds in this manner through a number of shifts in sorting principle. We used the 6-category WCST version, and the number of completed categories as a measure of executive function.

The *Vocabulary* subtest from the revised Wechsler Adult Intelligence Scale (WAIS) (Wechsler, 1997) was used as an estimate of pre-morbid intelligence. The participant is asked to explain the meaning of 40 words.

### 2.3 Statistics

All analyses were performed using IBM SPSS version 25. Group differences were analyzed with ANOVAs with partial eta squared as an estimate of effect size.

## 3 Results

The SLOF subscale scores were as follows: Physical functioning 23.8 (SD 1.8), Personal care skills 34.0 (SD 22.9), Interpersonal relationships 23.7 (SD 6.3), Social acceptability 35.3 (SD 20.4), Activities 48.4 (SD 7.5), and Work skills 20.5 (SD 5.1). These scores are highly similar to those from two other population based studies (Rocca et al., 2018; Mucci et al., 2014).

The neurocognitive function of the SSD patients was significantly lower than that of the healthy controls on all assessments, with very large effect sizes (Table 2). There was considerable diversity within each group, as subgroups of patients scored higher than the control mean and subgroups of controls scored lower than the patient mean, particularly on tests of working memory, verbal learning and memory, and executive function (Table 2).

**Table 2:** Neurocognitive function of the SSD patients (n = 291) and healthy controls (n = 302) and overlap in neurocognitive function between patients and controls.

Test	SSD patients Mean (SD)	Controls Mean (SD)	F	ɳ^2^	Patients > controls	Controls < patients
Speed of processing (TMT-A)	53.4 (27.3)	29.0 (10.6)	173.36⁎⁎⁎	0.26	*N* = 55 (18%)	*N* = 11 (4%)
Cognitive flexibility (TMT-B)	148.5 (87.4)	63.2 (25.6)	223.73⁎⁎⁎	0.31	*N* = 25 (9%)	*N* = 5 (2%)
Auditory working memory (LNS)	8.5 (2.7)	10.9 (2.9)	66.78⁎⁎⁎	0.12	*N* = 71 (24%)	*N* = 60 (20%)
Verbal learning (RAVLT-sum1–5)	37.6 (12.1)	52.4 (9.3)	195.31⁎⁎⁎	0.28	*N* = 38 (12%)	*N* = 20 (7%)
Retention verbal memory (RAVLT-7)	7.1 (3.4)	10.9 (3.1)	133.23⁎⁎⁎	0.21	*N* = 44 (15%)	*N* = 47 (16%)
Executive function (WCST Categories compl.)	2.7 (2.2)	5.0 (1.7)	149.63⁎⁎⁎	0.23	*N* = 69 (24.0%)	*N* = 60 (20%)
Executive function (WCST Total errors)	56.4 (24.9)	31.1 (20.9)	132.39⁎⁎⁎	0.21	–	–
Executive function (WCST Persev. resp.)	39.6 (28.8)	18.9 (12.8)	100.23⁎⁎⁎	0.17	–	–
Executive function (WCST Persev. errors)	33.2 (21.7)	15.9 (12.8)	112.48⁎⁎⁎	0.18	–	–
Attention and vigilance (CPT-IP Total d'prime)	0.4 (0.6)	1.2 (0.8)	178.83⁎⁎⁎	0.26	*N* = 24 (8%)	*N* = 27 (9%)
Premorbid function (WAIS Vocabulary)	38.6 (12.0)	47.5 (10.7)	89.34⁎⁎⁎	0.13	*N* = 72 (24%)	*N* = 27 (9%)

Neurocognitive test results in raw scores. F: group test. η^2^: effect size. Patients > controls: SSD patients with higher mean neurocognitive function than the mean level of controls. Controls < patients: healthy controls with lower mean neurocognitive function than the mean level of SSD patients.

⁎⁎⁎ *p* < .001.

The differences in clinical characteristics between the neurocognitively intact and impaired patients were as follows: Regarding the WSCT score, the cognitively intact group had a significantly lower PANSS negative symptom level (F -2.85, *p* < .01), a lower PANSS general pathology level (F -2.57, *p* < .05), and a lower PANSS total symptom level (F -2.76, p < .01). Regarding the WAIS Vocabulary score, the patient group with a higher score than the controls had a significantly lower PANSS negative symptom level (F -2.21, p < .05).

In order to avoid data redundancy, further analyses were done with only a selection of neurocognitive tests and their relationship to the SLOF subscales in the SSD patient group, for those participants who had completed all the relevant assessments. Table 3 depicts the mean neurocognitive scores of those patients who scored above or below the control mean, and the relationship of those scores to the SLOF domains. In general, those patients who scored highest on the neurocognitive tests had the highest level of practical functional skills. The effect sizes were highest for the tests of working memory and executive functions. There was no significant relationship between the neurocognitive test scores and the two SLOF dimensions that assess social skills.

**Table 3:** Neurocognitive (NC) scores across the functional domains (SLOF) in the patient group.

SLOF domain	NC test	Patients high N Mean SD	Patients low N Mean SD	F	ɳ^2^
Phys. functioning	TMT-B	19 24.8 (0.4)	161 23.8 (1.7)	7.12⁎⁎	0.04
Personal care skills	TMT-B	19 33.7 (1.9)	161 34.3 (25.7)	0.01	0.00
Interpersonal rel.	TMT-B	19 25.2 (4.8)	161 23.9 (6.5)	0.17	0.00
Social acceptability	TMT-B	19 34.4 (1.3)	161 35.6 (22.9)	0.50	0.00
Activities	TMT-B	19 52.6 (3.5)	161 48.3 (7.4)	6.27⁎	0.03
Work skills	TMT-B	19 23.8 (5.1)	161 20.2 (5.1)	8.55⁎⁎	0.05
Phys. functioning	LNS	52 24.1 (1.7)	126 23.8 (1.6)	1.36	0.01
Personal care skills	LNS	52 33.5 (2.6)	126 34.6 (29.1)	0.09	0.00
Interpersonal rel.	LNS	52 24.3 (6.6)	126 23.9 (6.4)	0.16	0.00
Social acceptability	LNS	52 34.2 (1.7)	126 36.0 (25.9)	0.26	0.00
Activities	LNS	52 51.0 (5.3)	126 47.7 (7.8)	8.00⁎⁎	0.04
Work skills	LNS	52 22.8 (5.0)	126 19.7 (5.0)	14.39⁎⁎⁎	0.08
Phys. functioning	RAVLT-1-5	30 24.6 (0.9)	154 23.7 (1.8)	6.72⁎	0.04
Personal care skills	RAVLT-1-5	30 33.8 (1.9)	154 34.3 (26.3)	0.01	0.00
Interpersonal rel.	RAVLT-1-5	30 25.2 (6.8)	154 23.7 (6.2)	1.41	0.01
Social acceptability	RAVLT-1-5	30 34.0 (2.0)	154 35.7 (23.4)	0.17	0.00
Activities	RAVLT-1-5	30 51.2 (3.6)	154 48.2 (7.7)	4.59⁎	0.03
Work skills	RAVLT-1-5	30 23.3 (5.0)	154 20.1 (5.1)	9.82⁎⁎	0.05
Phys. functioning	RAVLT-7	36 24.5 (1.0)	167 23.7 (1.8)	8.69⁎⁎	0.04
Personal care skills	RAVLT-7	36 33.9 (1.9)	167 32.1 (4.1)	0.01	0.00
Interpersonal rel.	RAVLT-7	36 25.5 (6.4)	167 23.4 (6.4)	3.50	0.02
Social acceptability	RAVLT-7	36 33.9 (2.7)	167 33.8 (1.8)	0.20	0.00
Activities	RAVLT-7	36 51.6 (3.8)	167 47.7 (7.9)	8.19⁎⁎	0.04
Work skills	RAVLT-7	36 23.6 (4.8)	167 19.8 (5.0)	14.93⁎⁎⁎	0.07
Phys. functioning	WCST	50 24.5 (1.1)	130 23.6 (1.9)	9.20⁎⁎	0.05
Personal care skills	WCST	50 40.2 (45.5)	130 32.1 (4.1)	4.13⁎	0.02
Interpersonal rel.	WCST	50 24.9 (6.6)	130 23.7 (6.2)	1.44	0.01
Social acceptability	WCST	50 33.9 (2.4)	130 36.1 (25.4)	0.39	0.00
Activities	WCST	50 51.9 (4.6)	130 47.4 (7.7)	15.07⁎⁎⁎	0.08
Work skills	WCST	50 23.8 (5.1)	130 19.5 (4.6)	29.04⁎⁎⁎	0.14
Phys. functioning	Vocabulary	48 24.1 (1.6)	133 23.8 (1.8)	1.17	0.01
Personal care skills	Vocabulary	48 32.7 (4.1)	133 34.7 (28.2)	0.24	0.00
Interpersonal rel.	Vocabulary	48 24.5 (6.3)	133 23.7 (6.5)	0.55	0.00
Social acceptability	Vocabulary	48 34.0 (2.4)	133 36.0 (25.1)	0.30	0.00
Activities	Vocabulary	48 50.4 (6.8)	133 47.8 (7.7)	4.48⁎	0.02
Work skills	Vocabulary	48 21.0 (5.3)	133 20.4 (5.2)	0.61	0.00

Patients high: Patients scoring higher than average controls in neurocognitive function. Patients low: Patients scoring lower than average controls in neurocognitive function. WCST: WCST Categories completed. F: group test. η^2^: effect size.

⁎⁎⁎ *p* < .001.

⁎⁎ *p* < .01.

⁎ *p* < .05.

## 4 Discussion

The first major finding from this study is that the neurocognitive function of our SSD participants and healthy controls are in accordance with numerous previous reports of a general cognitive deficit in the patient sample (e.g., Kahn and Keefe, 2013). Moreover, we found considerable overlap in neurocognitive function between the patient and the control group. In particular, a relatively high proportion of our SSD patients (15–24%) obtained test results higher than the control mean for indices of working memory, verbal learning and memory, and executive function. Similarly, a large minority of healthy controls (16–20%) scored below the patient mean on the same tests. These numbers correspond to previous findings of neurocognitive heterogeneity in SSD samples (Fioravanti et al., 2005; Joyce and Roiser, 2007; Shmukler et al., 2015; Weinberg et al., 2016. However, our participants are outpatients, and this group may display a better neurocognitive function level than inpatients do (Fioravanti et al., 2005). Studies of inpatients are likely to find smaller overlaps between patient and control samples.

Moreover, neurocognitive heterogeneity may be related to illness variables such as symptom level differences. In particular, a high level of psychosis symptoms has been found in the most cognitively impaired groups (Shmukler et al., 2015; Weinberg et al., 2016). We have previously reported significantly better cognitive function in remitted compared to non-remitted SSD patients (Helldin et al., 2006). Then we found statistically significant, but few and small symptom level differences in the cognitively intact and impaired patient groups. The relevant illness variables were the negative and general pathology symptoms, suggesting that patients exhibiting these clinical characteristics suffer the added disadvantage of reduced neurocognitive function.

This neurocognitive diversity, and the relationship to clinical variables, probably has significant implications for treatment response and real-world outcome in our participants. Compared to their unimpaired peers, the SSD patients that are most cognitively disadvantaged have demonstrated low psychosis symptom treatment response (Kennedy et al., 2015) and vocational ability (Lystad et al., 2016). Moreover, previous studies of the present SSD sample have shown that the subgroup with the poorest neurocognitive ability is at greater risk for somatic illness (Moradi et al., 2018) and premature death (Helldin et al., 2015) compared to patients with better cognitive ability.

In the present study, we extended our previous findings to the everyday functional skills area. We found that the SSD group with the lowest neurocognitive function scores had significantly lower levels of physical functioning and were significantly less able to perform practical skills necessary for personal grooming and care, general activities of daily life, and job-related tasks. This effect was found for several of the neurocognitive domains measured, although the relationship between indices of executive function and mental flexibility and the SLOF scores was the strongest. In a large sample of community dwellers with schizophrenia, a composite score of neurocognitive function was a stronger predictor of the SLOF composite score than symptom level, personal coping resources, and engagement with mental health services (Galderisi et al., 2014). In a largely male outpatient sample, composite neurocognitive function was related to practical life skills, but not social skills, of the SLOF (Strassnig et al., 2015). We have extended this finding to a gender-balanced sample.

Regarding specific cognitive domains, the importance of executive functions for everyday task performance has been noted by others (Kurtz and Wexler, 2006) in a predominantly male sample. We found the same association in a larger, gender mixed sample, suggesting that treatment of executive function deficits should be a priority in cognitive remediation programs aimed at increasing real-life functioning. However, the effect of executive function remediation on functional outcome in SSD may depend upon the baseline neurocognitive level of the participants, in that those who started out with the highest cognitive function level benefited the most from the cognitive intervention program (Vita et al., 2013). A limitation of the current study is that our sample size was too small to permit separate analyses of the neurocognitive and SLOF score associations in cognitively preserved and impaired participants. Therefore, we have no way of determining which our participants could be expected to benefit most from efforts to increase neurocognitive and real-world function.

We found no significant associations between neurocognitive function and social function skills. This is in accordance with some studies (Addington et al., 2005; Strassnig et al., 2015) and in contrast to others (Fu et al., 2017). Different ways of assessing social function may account for these divergent findings. Further, tests of social cognition could reveal significant relationships with interpersonal and social function. The fact that we did not assess social cognition is a limitation. Recently, we demonstrated that our SSD sample is characterized by significantly lower levels of positive affectivity than what is common in the healthy population (Mohn et al., 2018). This indicates that our participants are less likely to show enthusiasm, interest, and approach behavior in general. Possibly, this personality trait, and not neurocognitive function, may be relevant for social function.

The significant relationship between neurocognitive capacity and practical and work skills suggest that these functional domains in particular could benefit from cognitive remediation. Efforts to improve social function skills though cognitive training strategies targeting abilities required for developing and maintaining social relationships, such as attention span, learning, and mental flexibility, may not have the desired effects. A better strategy for improving social skills may be through treating negative symptoms of psychosis (Strassnig et al., 2015).

The clinical implication of our recent studies is that several outcome variables, such as remission potential, general health and mortality, and practical functioning skills, are related to differences in neurocognitive function in SSD. The large differences in cognitive performance within the patient group indicate a clear need for individualized treatment. Current treatment is dominantly based on symptom activity, although cognitive impairment is well-established as a core domain of schizophrenia. The importance of mean level neurocognitive performance for symptomatic and functional outcome is demonstrated in a large number of scientific reports. Here, we have linked neurocognitive heterogeneity to functional outcome differences, and suggest that personalized treatment with emphasis on practical daily skills may be of great significance especially for those with large baseline cognitive deficits. Such efforts are imperative not only in order to reduce personal suffering and increase quality of life for the patients, but also in order to reduce the enormous society level economic costs of functional deficits (Harvey and Strassnig, 2012).

Based on these findings, we recommend that neurocognitive assessment should be performed as early as possible after illness onset. Early identification of cognitive risk factors for poor real-life functional outcome is necessary in order to alert the clinical and rehabilitation services about patients in need of extra care. This additional strategy could move the field of rehabilitation further, as traditional anti-psychosis treatment has not significantly improved rates of independent living, social relations, and work-force participation in these patients.

### 4.1 Strengths and limitations

Our major strengths are the following: The population-based approach provides a naturalistic sample of community dwellers that are expected to manage several basic real-life function skills. Second, the self-reported level of drug and alcohol use was very low, and we are confident that our results reflect illness variables and are not artifacts of significant ongoing substance abuse. Third, our sample consists of both genders, increasing the ecological validity of our findings.

Major limitations are first that our sample was ethnically homogeneous, and our data may not generalize to ethnically and linguistically heterogeneous groups.

Second, our patient sample represented the entire duration of illness spectrum. Our aim was to provide a first description of the associations between neurocognitive and functional outcome in our participants. In the future, we will investigate this relationship in subgroups of patients based on their symptom severity and chronicity level.

Third, there were several different clinicians involved in the neuropsychological assessments, possibly generating a reliability problem. However, the group differences were in the expected direction and similar to those of other studies. Hence, we do no suspect biased neurocognitive results due to multiple testers.

Fourth, we used the 6-category WCST version. This program may generate a ceiling effect in the control group. Possibly, the group difference in executive function would be even larger had we used a more difficult test. Despite this limitation, however, the WCST score was related to all four SLOF subscales assessing practical skills.

Fifth, we did not obtain SLOF scores from the healthy control group. Therefore, we have no way of determining whether the neurocognitive and functional level relationship is unique to SSD patients, or if it occurs in other clinical groups and healthy individuals as well.

Sixth, we relied on self-report for information about the participants' drug and alcohol use. Results from a blood or urine sample would have been more valid. Therefore, some substance use could have been undetected. However, as has been shown by others in much larger samples (Harvey et al., 2020), neurocognitive function may be similar in drug-free and drug-using SSD groups.

## Funding

This study was funded by the Regional Health Authority, VG Region, Sweden, as part of their regular batch of funding for research and development activities. The funding body had no impact on the data analyses or manuscript preparation.

## CRediT authorship contribution statement

**Lars Helldin:** Conceptualization, Data curation, Funding acquisition, Methodology, Project administration, Supervision, Writing - review & editing. **Christine Mohn:** Methodology, Writing - review & editing. **Anna-Karin Olsson:** Data curation, Methodology, Writing - review & editing. **Fredrik Hjärthag:** Conceptualization, Funding acquisition, Methodology, Supervision, Writing - review & editing.

## Declaration of competing interest

The authors report no conflicts of interest.
